# Risk of colorectal cancer by family history of both colorectal carcinomas and colorectal polyps: a nationwide cohort study

**DOI:** 10.1002/cac2.70059

**Published:** 2025-09-02

**Authors:** Yuqing Hu, Elham Kharazmi, Qunfeng Liang, Hermann Brenner, Jan Sundquist, Kristina Sundquist, Mahdi Fallah

**Affiliations:** ^1^ Risk Adapted Prevention Group Division of Primary Cancer Prevention German Cancer Research Center (DKFZ) Heidelberg Germany; ^2^ Medical Faculty Heidelberg Heidelberg University Heidelberg Germany; ^3^ Center for Primary Health Care Research Lund University Malmo Sweden; ^4^ Division of Clinical Epidemiology and Aging Research German Cancer Research Center (DKFZ) Heidelberg Germany; ^5^ University Clinic Primary Care Region Skane Sweden

**Keywords:** colorectal cancer, colorectal polyp, family history, early‐onset colorectal cancer

## Abstract

**Background:**

The increased risk of colorectal cancer (CRC) associated with family history of both colorectal in situ or invasive carcinomas (Stage 0 to IV) and colorectal polyps is attributed solely to family history of CRC, resulting in an underestimation of the actual risk. We aimed to assess the association between overall and early‐onset CRC (EOCRC) risk and family history of both colorectal carcinomas and polyps.

**Methods:**

We conducted a nationwide cohort study leveraging Swedish family‐cancer datasets with follow‐up from 1964 to 2018. Standardized incidence ratios (SIRs) were calculated to estimate the risk of CRC and EOCRC among individuals with a family history of both colorectal polyps and carcinomas.

**Results:**

We followed up 13,432,205 individuals for up to 54 years. The risk of overall CRC was 2.2 times increased in individuals with 1 first‐degree relative (FDR) with one‐time polyp diagnosis and an additional FDR with carcinoma (95% CI = 2.1‐2.3; EOCRC SIR = 2.9 [95% CI = 2.4‐3.4]). The risk was significantly higher in individuals with 1 FDR with repeated polyp diagnoses (≥2 times) and an additional FDR with carcinoma (overall SIR = 2.9 [95% CI = 2.7‐3.1]; EOCRC SIR = 5.4 [95% CI = 3.9‐6.4]). A similar risk was observed in individuals with ≥2 FDRs with one‐time polyp diagnosis and an additional FDR with carcinoma (overall SIR = 2.9 [95% CI = 2.4‐3.4]; EOCRC SIR = 5.3 [95% CI = 3.0‐8.6]). Individuals with ≥2 FDRs with repeated polyp diagnoses and an additional FDR with carcinoma had a 5.0‐fold overall risk (95% CI = 4.3‐5.7) and a 13.8‐fold EOCRC risk (95% CI = 9.7‐20.1). Younger age at polyp/carcinoma diagnoses, and more relatives with polyps and carcinomas were associated with higher CRC risk.

**Conclusions:**

Individuals with a family history of both colorectal polyps and carcinomas are at significantly increased risk of CRC, especially EOCRC. The risk increased with frequent polyp diagnoses, younger age at first polyp/carcinoma diagnoses, and the number of relatives with polyps/carcinomas. This study highlights the importance of considering both colorectal polyps and carcinomas in family history when assessing CRC risk. These findings could supplement current screening guidelines.

**List of Abbreviations**
CI: confidence interval
CRC: colorectal cancer
EOCRC: early‐onset colorectal cancer
FDR: first‐degree relative
SDR: second‐degree relative
ICD: international classification of diseases
IBD: inflammatory bowel disease
HNPCC: hereditary nonpolyposis colorectal cancer
SIR: standardized incidence ratio

## 1 BACKGROUND

Colorectal cancer (CRC) ranks as the third most common malignancy and the second leading cause of cancer‐related mortality globally [1, 2]. While CRC screening programs for individuals aged 50 and above have contributed to a decrease in the incidence of late‐onset CRC by approximately 50% in the United States [3, 4], the incidence of early‐onset CRC (EOCRC) is on the rise and remains a global concern [5]. Identifying risk factors associated with CRC and EOCRC is crucial in developing risk‐adapted screening recommendations for the prevention and early detection of EOCRC. A family history of CRC, especially in first‐degree relatives (FDRs), has been identified as a significant risk factor for the development of CRC [6]. Consequently, individuals with such a family history are recommended to undergo earlier CRC screening [7]. A family history of colorectal polyps has likewise been associated with an elevated risk of CRC incidence [8], but there is inconsistency in the way these risk factors are considered (if any) in the screening guidelines.

The adoption of colonoscopy screening programs at the national and regional levels has led to a reduction in the incidence of CRC [4]. In contrast, there has been an increase in the detection of colorectal polyps [9]. Previous studies have investigated the association between CRC risk and family history of either CRC or polyps [6, 8], but there is a gap in our understanding of the family history of both colorectal polyps and the family history of colorectal carcinoma in situ (Stage 0) or CRC (Stage I to IV). While a previous study identified a synergistic association between a family history of both polyps and carcinomas, the sample sizes were relatively small, with 17 cases in the group with one FDR with carcinoma and ≥2 FDRs with polyps, and 15 cases in the group with one FDR with polyp and ≥2 FDRs with CRC [8]. The limited sample sizes also prevented the previous study from having detailed subgroup analyses by the youngest age at polyp detection and the number of relatives with polyps and carcinomas. Additionally, the study neither differentiated between one‐time and repeated polyp diagnoses (≥2 times) in the family, which has been reported as an important risk factor [10], nor considered second‐degree relatives (SDRs).

Therefore, to further investigate the association between the family history of both polyps and carcinomas and the risk of CRC, we leveraged the Swedish family‐cancer datasets, including over 13 million individuals followed up to 54 years between 1964 and 2018. As we have previously shown that family history of colorectal carcinoma in situ and family history of invasive CRC are associated with similar increased risk in other close relatives [11], in this study, we used the term family history of “colorectal carcinoma” as a representative of family history of either colorectal carcinoma in situ or invasive CRC. Colorectal carcinoma in situ, also known as Stage 0 CRC, refers to abnormal cells found in the innermost layer of the colon or rectum (the mucosa) that have the potential to become cancerous. These cells are confined to mucosa and have not invaded deeper layers of the colon or rectum wall or spread to other parts of the body. However, for the outcome, in this study we considered only overall invasive CRC (all ages) and EOCRC (diagnosed before age 50). We aimed to assess the association between CRC risk and family history of both carcinoma and polyp by the number of FDRs and SDRs with carcinoma and polyp, the youngest age at first polyp and carcinoma diagnosis, and the frequency of polyp diagnosis in relatives, with the overall aim of providing evidence for risk‐adapted screening.

## 2 MATERIALS AND METHODS

This study leveraged the Swedish family‐cancer datasets [12], which are the largest of their kind globally. Four nationwide datasets were linked using a unique pseudonymized national identification number: The Multi‐generation Register, National Patient Register, Swedish Cancer Registry, and Statistics Sweden. The Multi‐generation Register database registers children born in Sweden since 1932, along with their parents, providing genealogic (pedigree) data for the entire country. Data on cancer in FDRs and SDRs were extracted from this database through record linkage with cancer registry data. Information on medical records related to colorectal polyps was extracted from the National Patient Register, which contains information on all clinical visits of Swedish residents (inpatient visits since 1964 and outpatient specialty visits since 2000). The Swedish Cancer Registry has recorded patients with colorectal carcinomas since 1958 using the 7th Revision of the International Classification of Diseases (ICD‐7) codes and future revisions. Statistics Sweden provides vital information about individuals’ births, deaths, migration records, and other socioeconomic measures for the entire study period. These datasets are updated periodically, with the update in 2020 including over 13 million individuals followed up to the end of 2018 and an overall completeness of cancer data estimated at 96% or higher [13].

Our study included individuals who were born since 1932 and their parents, who resided in Sweden between January 1964 and December 2018, and had at least one known FDR in the genealogical dataset. Individuals diagnosed with inflammatory bowel disease (IBD) or hereditary non‐polyposis CRC (HNPCC) (*n* = 144,503) were excluded from the study. Follow‐up for each individual in our database started from the beginning of 1964, their birth year, or their immigration year, whichever occurred last. Follow‐up terminated when the individual was diagnosed with CRC, emigrated, passed away, or 31 December 2018, whichever occurred first.

Data pertaining to patients with colorectal carcinoma were obtained from the Swedish Cancer Registry dataset using ICD‐7 codes 153 and 154, with the exclusion of code 154.1 for the anus. The diagnosis of colorectal polyps was extracted from the National Patient Register in accordance with the ICD‐7, ICD‐8, ICD‐9, and ICD‐10 coding systems (Supplementary Table S1). If a person had several diagnoses of colorectal polyp and carcinoma in situ or CRC, only the diagnosis of the most advanced tumor was considered in the analysis.

Standardized incidence ratios (SIRs) were calculated to report the familial risk of CRC, including the risk of overall and EOCRC as the main outcomes, for individuals with a family history of both colorectal carcinomas and polyps. These calculations were adjusted for age (5‐year age groups), sex, calendar period (ranging from 1964 to 2018 in 5‐year intervals), region (including large cities, small cities in south Sweden, and small cities in north Sweden), history of diabetes mellitus, socioeconomic status (categorized as blue‐collar worker, white‐collar worker, farmer, self‐employed, professional, or other/unspecified), and inpatient and outpatient visits to specialty clinics due to obesity, alcoholism and chronic obstructive pulmonary disease (as a proxy for heavy smoking). A subgroup analysis was also performed for individuals aged <40 years and 40‐49 years at the time of CRC diagnosis, to explore potential age‐specific differences for EOCRC. Expected cases were calculated from strata‐specific person‐years in individuals with a certain family history of both polyps and carcinomas, multiplied by strata‐specific incidence rates in those without any family history of carcinomas or polyps. The 95% confidence intervals (CIs) of SIRs were calculated assuming a Poisson distribution.

SIR calculations were stratified by the number of relatives with a family history of both polyps and carcinomas, the frequency of polyp diagnosis (one‐time polyp diagnosis and ≥2 times polyp diagnoses at least 12 months apart), and the youngest age at polyp and carcinoma diagnoses (<60 and ≥60 years). All analyses were conducted using SAS software, version 9.4 (SAS Institute Inc., Cary, NC, USA). The forest plots were generated using R (version 4.3.2; R Foundation for Statistical Computing, Vienna, Austria). We followed the European Society for Medical Oncology (ESMO) Guidance for Reporting Oncology Real‐World evidence (GROW) during the writing of this manuscript [14].

## 3 RESULTS

This study included 13,432,205 individuals with at least one known FDR in our database. Of these participants, 51% (6,850,425) were men. The median follow‐up period was 31 years (25^th^ and 75^th^ percentiles: 13 and 49 years), during which 1.4% (188,070) patients with CRC were identified. Among the 12.5% (1,674,543) individuals with a family history of colorectal carcinomas (in situ or CRC), irrespective of a family history of polyps, 19.7% (329,690) were identified with a family history of both colorectal polyps and carcinomas.

In subjects with a familial history of polyps and an additional FDR diagnosed with either CRC or carcinoma in situ, we found a positive association between the familial risk of CRC and both the number of FDRs diagnosed with polyps and the frequency of polyp diagnosis among relatives (Table 1). Compared to individuals without any family history, those with 1 FDR with carcinoma and another FDR with a history of one‐time polyp diagnosis had a 2.2‐fold increased risk of CRC (95% CI = 2.1‐2.3). The risk escalated to 2.9‐fold (95% CI = 2.7‐3.1) in individuals with 1 FDR with carcinoma and another FDR with repeated (≥2 times) polyp diagnoses, a risk comparable to that in individuals with 1 FDR with carcinoma and ≥2 FDRs with one‐time polyp diagnosis (SIR = 2.9, 95% CI = 2.4‐3.4). The risk was significantly higher, at 5.0‐fold (95% CI = 4.3‐5.7), in individuals with 1 FDR with carcinoma and ≥2 FDRs with repeated polyp diagnoses. For individuals who have only SDR(s) with polyps and another FDR with carcinoma, the increased risk was mostly between 1.5‐fold and 2.0‐fold (Supplementary Table S2). However, in individuals with ≥2 SDRs with repeated polyp diagnoses and an additional FDR with carcinoma, a significantly higher SIR of 5.0 (3.6‐6.6) was noted. We found an increasing trend in risk of CRC with decreasing age at diagnosis in relatives diagnosed with polyps and carcinomas although some of the associated 95% CIs were overlapping (Table 1).

**TABLE 1:** Risk of CRC in relatives of patients diagnosed with colorectal polyp and additional FDR(s) with in situ or invasive colorectal carcinoma.

FDRs with carcinoma^a^	Relatives with polyp	Frequency of polyp diagnosis	Youngest age at polyp diagnosis (years)	Youngest age at carcinoma diagnosis (years)	No. of observed CRC patients	SIR^b^	95% CI
0	0 FDR/SDR	NA	NA	NA	142,234	Reference	Reference
1	0 FDR/SDR	NA	NA	All ages	15,521	**1.5**	1.5‐1.6
	≥1 FDR/SDR	≥1	All ages	All ages	3,670	**2.3**	2.2‐2.3
	1 FDR only	1	All ages	All ages	1,667	**2.2**	2.1‐2.3
			<60	<60	477	**3.1**	2.8‐3.4
				≥60	141	**2.5**	2.1‐2.9
			≥60	<60	401	**2.0**	1.8‐2.2
				≥60	648	**1.8**	1.7‐2.0
		≥2	All ages	All ages	602	**2.9**	2.7‐3.1
			<60	<60	166	**3.8**	3.2‐4.4
				≥60	77	**3.3**	2.6‐4.2
			≥60	<60	150	**2.9**	2.5‐3.4
				≥60	209	**2.3**	2.0‐2.7
	≥2 FDRs only	1	All ages	All ages	135	**2.9**	2.4‐3.4
			<60	<60	46	**5.1**	3.7‐6.8
				≥60	7	2.1	0.8‐4.5
			≥60	<60	41	**3.2**	2.3‐4.4
				≥60	41	**1.9**	1.3‐2.5
		≥2	All ages	All ages	185	**5.0**	4.3‐5.7
			<60	<60	71	**8.9**	6.9‐10.9
				≥60	17	**5.7**	3.3‐9.1
			≥60	<60	54	**5.9**	4.4‐7.6
				≥60	43	**2.5**	1.8‐3.4
≥2	0 FDR/SDR	NA	NA	All ages	1,306	**2.1**	2.0‐2.3
	≥1 FDR/SDR	≥1	Any	All ages	670	**3.7**	3.4‐4.0
	1 FDR only	1	All ages	All ages	218	**3.2**	2.8‐3.7
			<60	<60	67	**4.0**	3.1‐5.0
				≥60	12	**3.7**	1.9‐6.2
			≥60	<60	95	**3.4**	2.7‐4.1
				≥60	44	**2.3**	1.7‐3.2
		≥2	All ages	All ages	92	**3.7**	3.0‐4.6
			<60	<60	31	**4.8**	3.2‐6.8
				≥60	3	2.9	0.5‐8.0
			≥60	<60	37	**3.3**	2.3‐4.5
				≥60	21	**3.6**	2.2‐5.5
	≥2 FDRs only	1	All ages	All ages	30	**4.2**	2.8‐6.0
		≥2	All ages	All ages	41	**6.3**	4.5‐8.6

Abbreviations: CI, confidence interval; CRC, colorectal cancer; FDR, first‐degree relative; SDR, second‐degree relative; SIR, standardized incidence ratio.

^a^ Including colorectal in situ and invasive carcinoma (Stage 0 to IV).

^b^ SIR adjusted for age, sex, calendar year, region, history of diabetes mellitus, socioeconomic status, and inpatient and outpatient visits to specialty clinics due to obesity, alcoholism and chronic obstructive pulmonary disease. Bold SIR indicates statistically significant (95% CIs did not include 1.00).

For individuals with a familial history of polyps who also had ≥2 FDRs with carcinomas, there was a further increase in familial CRC risk (Table 1). Individuals with one FDR with one‐time polyp diagnosis and ≥2 FDRs with carcinomas showed a 3.2‐fold increased risk (95% CI = 2.8‐3.7) compared to those without any family history. The risk was 3.7‐fold (95% CI = 3.0‐4.6) in individuals with one FDR diagnosed with repeated polyp diagnoses and ≥2 FDRs with carcinomas. Individuals with ≥2 FDRs diagnosed with carcinomas and ≥2 FDRs with one‐time polyp diagnosis had a 4.2‐fold increased risk (95% CI = 2.8‐6.0), whereas those with ≥2 FDRs diagnosed with carcinomas and ≥2 FDRs diagnosed with repeated polyp diagnoses had a much higher risk of 6.3‐fold (95% CI = 4.5‐8.6). We found an increased risk when a relative was diagnosed with carcinoma or polyp at a younger age. In individuals who had only SDR(s) with polyps and ≥2 FDRs with carcinomas, the risk of CRC ranged between 3.0‐fold and 5.8‐fold across all groups (Supplementary Table S2).

**FIGURE 1:**
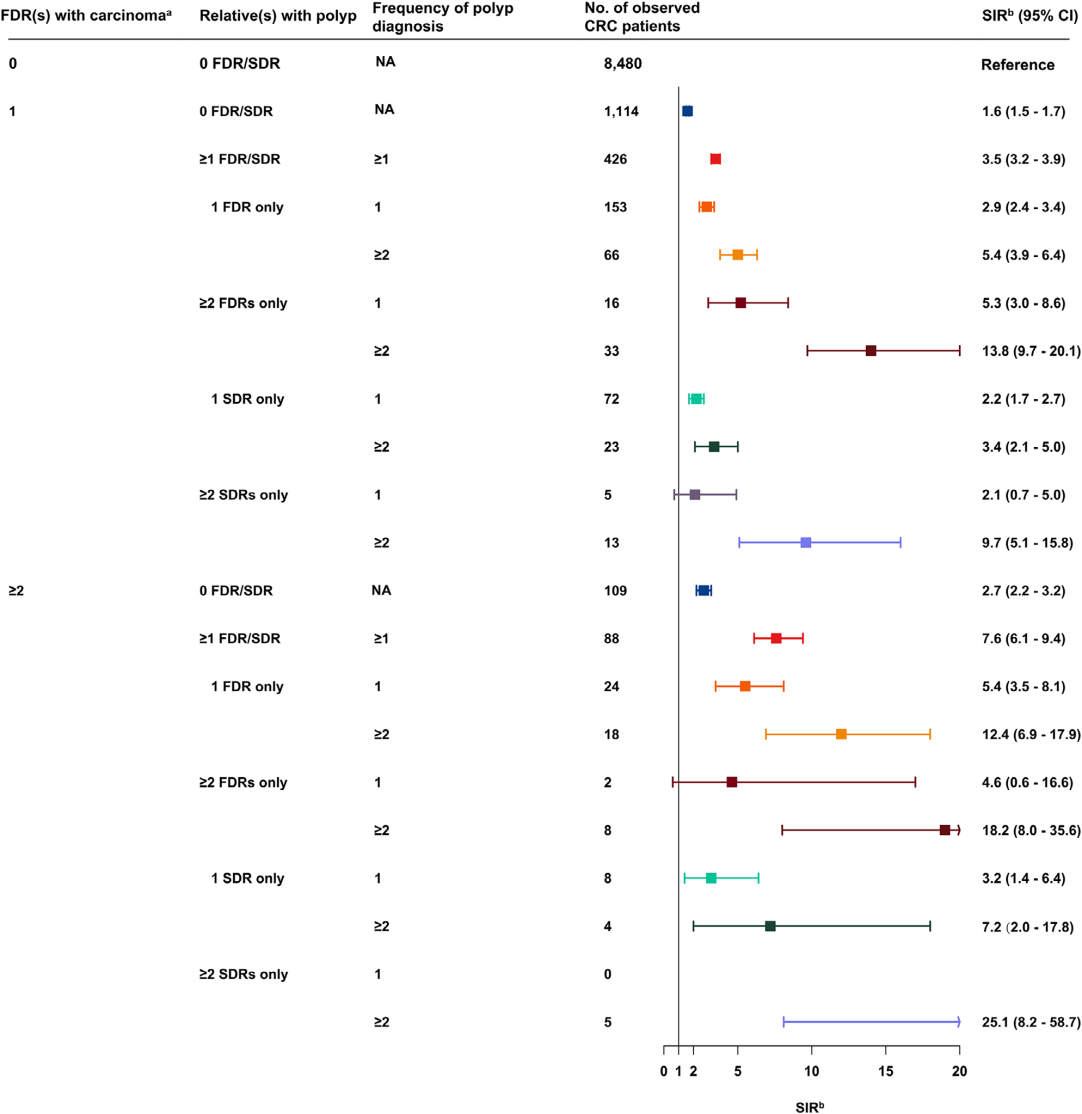
Risk of EOCRC in relatives of patients diagnosed with colorectal polyp and additional FDR(s) with colorectal in situ or invasive carcinoma. ^a^Including in situ and invasive colorectal carcinoma (Stage 0 to stage IV). ^b^SIR adjusted for age, sex, calendar year, region, history of diabetes mellitus, and socioeconomic status, and inpatient and outpatient visits to specialty clinics due to obesity, alcoholism and chronic obstructive pulmonary disease. Abbreviations: CI, confidence interval; CRC, colorectal cancer; EOCRC, early‐onset colorectal cancer; FDR, first‐degree relative; SDR, second‐degree relative; SIR, standardized incidence ratio.

In individuals with a family history of polyps and an additional FDR with carcinoma, the risk of EOCRC increased with the increasing number of FDRs with polyps and increasing frequency of polyp diagnosis (Figure 1). Individuals with 1 FDR with one‐time polyp diagnosis and an additional FDR with carcinoma exhibited a 2.9‐fold (95% CI = 2.4‐3.4) increase in EOCRC risk compared to those without any family history. This risk elevated to 5.4‐fold (95% CI = 3.9‐6.4) for individuals with 1 FDR with repeated polyp diagnoses and an additional FDR with carcinoma, similar to individuals with ≥2 FDRs with one‐time polyp diagnosis and an additional FDR with carcinoma (SIR = 5.3, 95% CI = 3.0‐8.6). Individuals with ≥2 FDRs with repeated polyp diagnoses and an additional FDR with carcinoma had a remarkable 13.8‐fold risk of CRC (95% CI = 9.7‐20.1). When individuals had only SDR(s) with polyps and an additional FDR with carcinoma, the risk of EOCRC was 2.2‐fold (95% CI = 1.7‐2.7) for individuals with 1 SDR with one‐time polyp diagnosis, 3.4‐fold (95% CI = 2.1‐5.0) for individuals with 1 SDR with repeated polyp diagnoses, 2.1‐fold (95% CI = 0.7‐5.0) for individuals with ≥2 SDRs with one‐time polyp diagnosis, and 9.7‐fold (95% CI = 5.1‐15.8) for individuals with ≥2 SDRs with repeated polyp diagnoses.

We found an increased risk of EOCRC in individuals with ≥2 FDRs with carcinomas and a family history of polyps (Figure 1). Individuals with 1 FDR with one‐time polyp diagnosis and ≥2 FDRs with carcinomas had 5.4‐fold (95% CI = 3.5‐8.1) increased risk of EOCRC. This risk increased to 12.4‐fold (95% CI = 6.9‐17.9) for individuals with 1 FDR with repeated polyp diagnoses and ≥2 FDRs with carcinomas, and to 18.2‐fold (95% CI = 8.0‐35.6) in individuals with ≥2 FDRs with repeated polyp diagnoses and ≥2 FDRs with carcinomas. When considering individuals with only SDR(s) with polyps and ≥2 FDRs with carcinomas, the risk was 3.2‐fold (95% CI = 1.4‐6.4) for individuals with 1 SDR with one‐time polyp diagnosis, 7.2‐fold (95% CI = 2.0‐17.8) for individuals with 1 SDR with repeated polyp diagnoses, and 25.1‐fold (95% CI = 8.2‐58.7) for individuals with ≥2 SDRs with repeated polyp diagnoses. In subgroup analyses, the associations remained consistent among individuals aged <40 years and those aged 40‐49 years (Supplementary Table S3, Supplementary Table S4).

In individuals with a family history of polyps and SDR(s) with carcinomas, the risk increased with the number of SDR(s) with carcinomas, with a profound increase in the risk of EOCRC (Table 2). For individuals with 1 FDR diagnosed with polyp once, the risk of EOCRC was 1.8‐fold (95% CI = 1.4 ‐ 2.2) when combined with 1 SDR with carcinoma, and the risk increased to 2.5‐fold (95% CI = 1.4 ‐ 4.3) when combined with ≥2 SDRs with carcinomas.

**TABLE 2:** Risk of CRC in relatives of patients diagnosed with colorectal polyp and additional SDR(s) with colorectal in situ or invasive carcinoma.

			Overall CRC			EOCRC		
SDRs with carcinoma^a^	Number of relatives with polyp	Frequency of polyp diagnosis	No. of observed CRC patients	SIR^b^	95% CI	No. of observed CRC patients	SIR^b^	95% CI
**0**	0 FDR/SDR	NA	142,234	Reference	Reference	8,480	Reference	Reference
**1**	1 FDR only	1	232	**1.5**	1.3‐1.7	65	**1.8**	1.4‐2.2
		≥2	75	**1.9**	1.5‐2.3	24	**2.5**	1.6‐3.8
	≥2 FDRs only	1	24	**2.5**	1.6‐3.7	2	1.4	0.3‐5.0
		≥2	24	**3.0**	1.9‐4.5	9	**9.6**	4.4‐17.5
	1 SDR only	1	185	**1.3**	1.1‐1.5	77	**1.2**	1.0‐1.6
		≥2	48	**1.6**	1.2‐2.1	14	1.1	0.5‐1.7
	≥2 SDRs only	1	23	**1.9**	1.2‐2.9	11	1.7	0.9‐3.1
		≥2	17	**2.2**	1.3‐3.6	11	**2.8**	1.4‐5.0
**≥2**	1 FDR only	1	22	**2.0**	1.2‐3.0	14	**2.5**	1.4‐4.3
		≥2	8	2.1	0.9‐4.2	4	2.3	0.6‐5.6
	≥2 FDRs only	1	2	3.1	0.4‐10.6	1	5.2	0.1‐27.5
		≥2	2	**8.1**	1.0‐28.6	1	6.9	0.2‐36.8
	1 SDR only	1	20	1.3	0.8‐2.0	12	1.2	0.6‐2.2
		≥2	8	1.7	0.7‐3.4	3	1.4	0.2‐3.3
	≥2 SDRs only	1	4	2.2	0.6‐5.7	3	2.6	0.5‐7.3
		≥2	1	0.7	0.1‐3.6	0	NA	NA

Abbreviations: CI, confidence interval; CRC, colorectal cancer; EOCRC, early‐onset colorectal cancer; FDR, first‐degree relative; SDR, second‐degree relative; SIR, standardized incidence ratio; NA, not applicable.

^a^ Including colorectal in situ and invasive carcinoma (Stage 0 to IV).

^b^ SIR adjusted for age, sex, calendar year, region, history of diabetes mellitus, and socioeconomic status, and inpatient and outpatient visits to specialty clinics due to obesity, alcoholism and chronic obstructive pulmonary disease. Bold SIR indicates statistically significant (95% CIs did not include 1.00).

When we changed the reference groups from individuals without any family history of polyp and carcinoma to individuals with certain family history of carcinomas, but no polyp, we still observed a significantly higher risk of overall CRC and EOCRC in those with both family history (Supplementary Figure S1 and Supplementary Figure S2). There was no change in the pattern of risk, which increased with the number of FDRs with polyps and the frequency of polyp diagnosis in FDRs. Compared with individuals with 1 FDR with carcinoma alone, the risk of overall CRC was 1.4‐fold (95% CI = 1.4‐1.5) and 1.9‐fold (95% CI = 1.7‐2.0) increased in individuals with an additional FDR with 1‐time or repeated polyp diagnoses, respectively. For the risk of EOCRC, the SIR was 1.8 (95% CI = 1.5‐2.1) in individuals with an additional FDR with a 1‐time polyp diagnosis and 3.2 (95% CI = 2.5‐4.0) in individuals with an additional FDR with repeated polyp diagnoses.

## 4 DISCUSSION

Our nationwide cohort study indicated an increased risk of CRC in individuals with a family history of both polyps and carcinomas *(in situ* or CRC) in different close family members. Younger age at polyp and carcinoma diagnosis, higher number of FDRs with polyps and carcinomas, and more repeated polyp diagnoses were associated with a higher risk of CRC. The association was more pronounced for the risk of EOCRC. In addition, our findings suggested a synergistic effect in individuals with at least 2 FDRs diagnosed with polyps (particularly repeated polyp diagnoses) and carcinomas. It is important to highlight that individuals with SDRs with polyps and carcinomas are also at increased risk.

Present guidelines advocate for earlier CRC screening in individuals with a family history of CRC compared to the general population [15, 16]. However, these guidelines often overlook the familial risk of CRC that can manifest itself as a family history of polyps. This occurs when lesions in relatives are detected early (e.g., through screening) and removed, thereby likely preventing the development of CRC. As a result, the “additional” increased risk due to family history of polyps in individuals with a family history of both polyps and carcinomas is often overlooked. Nevertheless, our findings suggested a significantly higher risk of CRC in individuals with a family history of both polyps and carcinomas than the risk in those with a family history of carcinomas alone. Therefore, it may be worthwhile developing personalized screening protocols for individuals with a familial history of both polyps and carcinomas, particularly those with a greater number of FDRs diagnosed with polyps and carcinomas, more frequent polyp diagnoses, and a younger age at diagnosis of polyp or carcinoma. Further research is warranted to elucidate the risk‐tailored starting age of screening in individuals with a family history of both polyps and carcinomas.

A more potent association was observed between the risk of EOCRC and the family history of both polyps and carcinomas. EOCRC has emerged as a global concern due to its rising incidence [17], advanced stage at diagnosis, and poorer prognosis [18]. The risk factors associated with EOCRC remain incompletely identified, and efforts are currently underway to develop risk‐adapted screening protocols aimed at reducing the number of EOCRC patients [19]. Our findings provide insights into the risk of EOCRC associated with detailed family history not only of colorectal carcinomas but also of polyps, and suggest that individuals with a familial history of both polyps and carcinomas are at a significantly elevated risk of EOCRC. This risk was particularly pronounced in individuals with ≥2 FDRs diagnosed with carcinomas and ≥2 FDRs with repeated polyp diagnoses, exhibiting an increase in risk of up to 18.2‐fold. For individuals with a familial history of both polyps and carcinomas, it may be crucial to initiate screening at an earlier age and maintain a more frequent screening interval to reduce the incidence and mortality associated with EOCRC. Recommending genetic counseling [20], as well as gut microbiome testing and analysis [21], for these high‐risk individuals warrants further investigation.

A previous study has identified a synergistic association between individuals with ≥2 FDRs with polyps and ≥2 FDRs with carcinomas, and the risk of CRC [8]. Our study further indicated that this synergistic association primarily stems from the relatives with repeated polyp diagnoses rather than one‐time polyp diagnosis. The risk of CRC was 3.1‐fold in individuals with ≥2 FDRs with carcinomas [8], 1.9‐fold in individuals with ≥2 FDRs with one‐time polyp diagnosis, and 2.4‐fold (95% CI = 2.2‐2.7) in individuals with ≥2 FDRs with repeated polyp diagnoses [22]. The risk increased to 4.2‐fold (95% CI = 2.8‐6.0) in individuals with ≥2 FDRs with one‐time polyp diagnosis and ≥2 FDRs with carcinomas, and 6.3‐fold in individuals with ≥2 FDRs with repeated polyp diagnoses and ≥2 FDRs with carcinomas. This pattern was more consistent for EOCRC. The frequency of polyp diagnosis in individuals and the subsequent colonoscopy surveillance represents a contentious topic among scientists and clinicians [23, 24]. Frequency of polyp diagnosis not only impacts the individuals’ health, but our findings suggest that repeated polyp diagnoses are also associated with an increased risk of CRC in relatives, particularly in those with a family history of both carcinomas and repeated polyp diagnoses in FDRs.

Our study also showed that a family history of both colorectal carcinomas and polyps in SDR(s) may be relevant for risk stratification. Compared with having only SDR(s) with polyps [10], individuals who also had SDR(s) with carcinomas demonstrated a higher familial risk of CRC. In the real‐world practice, while awareness of CRC risk in FDRs is relatively common, the Delphi Initiative for EOCRC International Management Guidelines reports that approximately 28% of patients with EOCRC have a family history of the disease [25]. However, awareness of polyp diagnosis in FDRs, and even more so in SDRs, is often significantly limited. Moreover, individuals with a family history of both polyps and colorectal carcinomas are less likely to be adequately informed about their risk due to the current lack of knowledge in this regard in literatures. This gap may lead to an underappreciation of familial CRC risk and suboptimal decisions regarding screening. Our findings aim to help close this gap by highlighting the highly elevated CRC risk associated with a family history of both polyps and colorectal carcinomas. In addition to the extensively studied factors such as polyp type, size, and number, our findings highlight the significance of the frequency of polyp diagnosis when considering the family history of polyps. A prior study has demonstrated that most polyps tend to remain stable or regress [26]. Future research could explore identifying polyps with growth potential and frequent occurrence, both in individuals and their relatives, and developing risk‐adapted screening strategies based on these findings.

Our study possessed several strengths. We reported the detailed familial risk of CRC in individuals with a family history of both polyps and carcinomas, leveraging the nationwide register‐based family‐cancer data, the largest of its kind in the world. A family history of both polyps and carcinomas is often regarded as just a family history of CRC, leading to an underestimation of the CRC risk and the need for an even earlier and more intensive screening in such individuals. Our study found a significantly higher CRC risk when both conditions are present in the family history compared to carcinomas alone, highlighting the need for increased attention. This high‐quality database significantly mitigated the influence of selection and recall bias and provided a large sample size and robust evidence to report the risk of CRC even in most of the detailed subgroups. We also demonstrated that the observed synergistic risk elevation in multiple FDRs with polyps and carcinomas probably comes from the impact of repeated polyp diagnoses in relatives, which merits future investigation. Furthermore, we incorporated SDR(s) with polyps and carcinomas into our calculation and found that some groups were at high risk and warrant further attention.

Some limitations should be acknowledged, as we were unable to directly adjust for certain lifestyle factors, such as obesity, smoking, physical activity, and alcohol use, due to a lack of data. However, in our main analyses, we adjusted for region of residence and inpatient and outpatient visits to specialty clinics due to chronic obstructive pulmonary disease (as a proxy for heavy smoking), obesity, and alcoholism, which may have partially accounted for these factors. Nonetheless, residual confounding cannot be entirely ruled out, and further studies with detailed lifestyle data are warranted. Although we adjusted for some relevant comorbidities such as diabetes, chronic obstructive pulmonary disease, obesity, alcohol abuse, and excluded individuals with IBD and HNPCC, detailed information on other conditions was not available. Given that our study focused on cancer risk (incidence rather than mortality), particularly CRC diagnosed before age 50, when serious comorbidities are less common, the impact of this limitation is likely to be trivial. Nevertheless, future studies should aim to include a broader range of comorbidities for more comprehensive adjustment.

We also lacked data on polyp characteristics, such as size, number, and histological classification, which limited our ability to evaluate the association between these factors in relatives and CRC risk. However, a previous study by Song *et al.* [8] found no significant differences in CRC risk based on the histological types of polyps in relatives. Given the considerable overlap between our study population and that of Song *et al.* [8], we believe our findings are applicable across various histological types of polyps in relatives. Further research is also warranted to explore whether other features of polyps in family, such as size or number, might be associated with CRC risk, particularly when combined with a family history of colorectal carcinomas. Although the absence of detailed information on polyp number, histology, and size is a limitation, it may more accurately reflect real‐world scenarios. In practice, individuals are unlikely to know these specific details about their relatives' polyps, but are more likely to be aware of whether their relatives have undergone a single colonoscopy with polyp removal or multiple procedures.

Additionally, we observed limited data in some subgroups, particularly those involving SDRs diagnosed with both polyps and carcinomas; as such, the corresponding results—with wide 95% CIs—are presented as exploratory and should be interpreted with appropriate caution. In addition, although individuals with IBD and HNPCC were excluded from the analysis, we were unable to identify and exclude those with familial adenomatous polyposis due to the lack of specific data in the registers. However, given the extremely low prevalence of this condition [27], any potential impact on the overall results is likely to be negligible. Moreover, although our study spans several decades during which screening practices have evolved—particularly with the broader adoption of colonoscopy—we believe these changes are unlikely to have significantly influenced our primary findings. In Sweden, there have been no national screening recommendations specifically aimed at individuals with a family history of polyps [28], reducing the chance of systematically increased surveillance in this group. Additionally, our prior sensitivity analyses [10], which excluded relatives with a one‐time polyp diagnosis following colonoscopy, yielded consistent results, suggesting that variations in screening intensity did not substantially alter the observed associations. Furthermore, we have adjusted for calendar period (from 1964 to 2018 in 5‐year intervals) in our analyses to mitigate the potential impact of technological and medical advancements over time in Sweden. Taken together, these considerations support the robustness of our results despite the temporal changes in medical practice.

Finally, as our study is based on Swedish nationwide data, our results should be most likely applicable to countries with rather similar genetic backgrounds, distributions of risk factors, CRC incidence rates, and healthcare systems. Nevertheless, the generalizability of the findings to other populations requires caution, and future studies in more diverse settings are warranted.

## 5 CONCLUSIONS

Our study identified that individuals with a family history of both polyps and carcinomas (in situ or CRC) in FDRs are at significantly increased risk of CRC, especially EOCRC. The risk increased with factors such as higher frequency of polyp diagnoses, younger age at polyp and carcinoma diagnoses, and the number of relatives with polyps and carcinomas. This study highlights the importance of considering both colorectal polyps and carcinomas in family history when assessing CRC risk, and these findings can complement current screening guidelines. Further investigations into the development of risk‐adapted screening strategies are warranted for individuals with a family history of both polyps and carcinomas.

## AUTHOR CONTRIBUTIONS

Mahdi Fallah and Elham Kharazmi conceived the study. Kristina Sundquist and Jan Sundquist provided the study material. Yuqing Hu designed the detailed protocol, performed the data analysis, and drafted the manuscript. Yuqing Hu, Elham Kharazmi, Qunfeng Liang, Hermann Brenner, and Mahdi Fallah interpreted the results. All authors have critically revised the manuscript and approved the final version. The corresponding author attests that all listed authors meet authorship criteria and that no others meeting the criteria have been omitted. Mahdi Fallah is the guarantor.

## CONFLICT OF INTEREST STATEMENT

All authors declare no conflicts, no support from any organization for the submitted work (other than that described above), no financial relationships with any organizations that might have an interest in the submitted work during the previous three years, and no other relationships or activities that could appear to have influenced the submitted work.

## ETHICS APPROVAL AND CONSENT TO PARTICIPATE

The study was approved by the Lund Regional Ethical Review Board (2012/795 and subsequent amendments). Written informed consent was not required, as only pseudonymized data were used and no personal information could be unmasked.

## Supporting information



Supporting Information

## Data Availability

This study made use of information from the Multi‐generation Register, Statistics Sweden, Swedish Cancer Registry as well as the Swedish National Patient Register. Data from these registers cannot be shared by study authors, however further information and relevant contact details can be found on: https://www.socialstyrelsen.se/en/statistics‐and‐data/registers/. Links (email addresses) for registers: https://www.socialstyrelsen.se/en/statistics‐and‐data/registers/national‐cancer‐register/ (cancerregistret@socialstyrelsen.se), https://www.socialstyrelsen.se/en/statistics‐and‐data/registers/national‐patient‐register/ (patientregistret@socialstyrelsen.se).
