# Supplementary material for: Risk of colorectal cancer by family history of both colorectal carcinomas and colorectal polyps: a nationwide cohort study
Source: Cancer Commun (Lond). 2025 Sep 2;45(11):1407–16. doi: 10.1002/cac2.70059 (PMC12629855; doi:10.1002/cac2.70059)
Supplement: Supplementary file 1 — Supporting Information [file CAC2-45-1407-s001.docx]

**Supplementary Materials**

**Risk of colorectal cancer by family history of both colorectal carcinomas and colorectal polyps: a nationwide cohort study**

Yuqing Hu^1,2^, Elham Kharazmi^3^, Qunfeng Liang^1,2^, Hermann Brenner^4^, Jan Sundquist^3,5^, Kristina Sundquist^3,5^, Mahdi Fallah^1,3,*^

^1^Risk Adapted Prevention Group, Division of Primary Cancer Prevention, German Cancer Research Center (DKFZ), Heidelberg, Germany.

^2^Medical Faculty Heidelberg, Heidelberg University, Heidelberg, Germany.

^3^Center for Primary Health Care Research, Lund University, Malmo, Sweden.

^4^Division of Clinical Epidemiology and Aging Research, German Cancer Research Center (DKFZ), Heidelberg, Germany.

^5^University Clinic Primary Care, Region Skane, Sweden.

^*^Corresponding author:

Mahdi Fallah; Risk Adapted Prevention (RAD) Group, Division of Primary Cancer Prevention, German Cancer Research Center (DKFZ), Im Neuenheimer Feld 581,

69120, Heidelberg, Germany; Tel.: +49 6221 42 3040; Fax: +49 6221 56 5231; Email: m.fallah@dkfz.de.

**Supplementary Table S1. ICD codes for colorectal polyp.**

| **ICD version** | **Register period** | **Codes** |
| --- | --- | --- |
| ICD-7 | 1964-1968 | 211 |
| ICD-8 | 1969-1986 | 211.3, 211D, 21.4, and 211E |
| ICD-9 | 1987-2005 | 569, 569A, 556.4, V12.72, V12H, 211.3, 211D, 211.4, and 211E |
| ICD-10 | 2006-2018 | K63.5, K62.1, K51.4, JFA15, JGA05, and D12 |

Abbreviations: ICD, International Classification of Disease.

**Supplementary Table S2. Risk of CRC in relatives of patients diagnosed with colorectal polyp and additional FDR(s) with colorectal *in situ* or invasive carcinoma.**

| FDRs with carcinoma^a^ | Relatives with polyp | Frequency of polyp diagnosis | Youngest age at polyp diagnosis  (years) | Youngest age at carcinoma diagnosis  (years) | No. of observed CRC patients | SIR^b^ | 95% CI |
| --- | --- | --- | --- | --- | --- | --- | --- |
| 0 | 0 FDR/SDR | NA | NA | NA | 142,234 | Reference | Reference |
| 1 | 1 SDR only | 1 | All ages | All ages | 610 | **1.6** | 1.5-1.8 |
|  |  |  | <60 | <60 | 469 | **1.7** | 1.5-1.8 |
|  |  |  |  | ≥60 | 39 | **1.4** | 1.0-1.9 |
|  |  |  | ≥60 | <60 | 59 | **1.9** | 1.5-2.5 |
|  |  |  |  | ≥60 | 43 | **1.4** | 1.1-1.9 |
|  |  | ≥2 | All ages | All ages | 122 | **1.7** | 1.4-2.0 |
|  |  |  | <60 | <60 | 87 | **1.6** | 1.3-1.9 |
|  |  |  |  | ≥60 | 10 | 1.8 | 0.8-3.1 |
|  |  |  | ≥60 | <60 | 12 | **2.7** | 1.4-4.6 |
|  |  |  |  | ≥60 | 13 | **2.2** | 1.2-3.7 |
|  | ≥2 SDRs only | 1 | All ages | All ages | 32 | **1.9** | 1.3-2.7 |
|  |  |  | <60 | <60 | 25 | **2.2** | 1.4-3.2 |
|  |  |  |  | ≥60 | 1 | 1.2 | 0.1-6.2 |
|  |  |  | ≥60 | <60 | 4 | 1.8 | 0.5-4.7 |
|  |  |  |  | ≥60 | 2 | 1.0 | 0.1-3.1 |
|  |  | ≥2 | All ages | All ages | 46 | **5.0** | 3.6-6.6 |
|  |  |  | <60 | <60 | 36 | **5.1** | 3.6-7.1 |
|  |  |  |  | ≥60 | 2 | 3.0 | 0.4-10 |
|  |  |  | ≥60 | <60 | 3 | **5.5** | 1.1-15 |
|  |  |  |  | ≥60 | 5 | **5.4** | 1.7-12 |
| ≥2 | 1 SDR only | 1 | All ages | All ages | 121 | **3.0** | 2.5-3.6 |
|  |  | ≥2 | All ages | All ages | 32 | **3.4** | 2.3-4.8 |
|  | ≥2 SDRs only | 1 | All ages | All ages | 15 | **4.2** | 2.4-6.9 |
|  |  | ≥2 | All ages | All ages | 18 | **5.8** | 3.4-9.2 |

^a^Including colorectal in situ and invasive carcinoma (Stage 0 to IV).

^b^SIR adjusted for age, sex, calendar year, region, history of diabetes mellitus, and socioeconomic status. Bold SIR indicates statistically significant (95% CIs did not include 1.00).

Abbreviations: CI, confidence interval; CRC, colorectal cancer; EOCRC, early-onset colorectal cancer; FDR, first-degree relative; SDR, second-degree relative; SIR, standardized incidence ratio; NA, not applicable.

**Supplementary Table S3. Risk of EOCRC (diagnosed before 40 years) in relatives of patients diagnosed with colorectal polyp and additional FDR(s) with colorectal *in situ* or invasive carcinoma.**

| FDRs with carcinoma^a^ | Relatives with polyp | Frequency of polyp diagnosis | No. of observed CRC patients | SIR^b^ | 95% CI |
| --- | --- | --- | --- | --- | --- |
| 0 | 0 FDR/SDR | NA | 2,591 | Reference | Reference |
| 1 | 0 FDR/SDR | NA | 315 | **1.6** | 1.4-1.8 |
|  | ≥1 FDR/SDR | ≥1 | 126 | **3.4** | 2.8-4.0 |
|  | 1 FDR only | 1 | 40 | **2.6** | 1.9-3.6 |
|  |  | ≥2 | 19 | **5.1** | 3.1-8.0 |
|  | ≥2 FDRs | 1 | 5 | **5.9** | 2.0-13.5 |
|  |  | ≥2 | 7 | **10.1** | 4.4-22.4 |
|  | 1 SDR only | 1 | 27 | **2.3** | 1.6-3.5 |
|  |  | ≥2 | 7 | **2.9** | 1.3-6.1 |
|  | ≥2 SDRs | 1 | 2 | 2.2 | 0.3-8.1 |
|  |  | ≥2 | 6 | **11.5** | 4.2-25.0 |
| ≥2 | 0 FDR/SDR | NA | 34 | **2.9** | 2.0-4.1 |
|  | ≥1 FDR/SDR | ≥1 | 24 | **7.3** | 4.7-10.1 |
|  | 1 FDR only | 1 | 7 | **5.6** | 2.3-11.6 |
|  |  | ≥2 | 5 | **11.5** | 3.7-26.8 |
|  | ≥2 FDRs | 1 | 0 | NA | NA |
|  |  | ≥2 | 0 | NA | NA |
|  | 1 SDR only | 1 | 1 | 1.4 | 0-7.9 |
|  |  | ≥2 | 3 | **18.5** | 3.8-54.1 |
|  | ≥2 SDRs | 1 | 0 | NA | NA |
|  |  | ≥2 | 2 | **39** | 4.5-134.6 |

^a^Including colorectal *in situ* and invasive carcinoma (Stage 0 to IV).

^b^SIR adjusted for age, sex, calendar year, region, history of diabetes mellitus, and socioeconomic status, and inpatient and outpatient visits to specialty clinics due to obesity, alcoholism and chronic obstructive pulmonary disease. Bold SIR indicates statistically significant (95% CIs did not include 1.00).

Abbreviations: CI, confidence interval; CRC, colorectal cancer; EOCRC, early-onset colorectal cancer; FDR, first-degree relative; SDR, second-degree relative; SIR, standardized incidence ratio; NA, not applicable.

**Supplementary Table S4. Risk of EOCRC (diagnosed between 40 and 49 years) in relatives of patients diagnosed with colorectal polyp and additional FDR(s) with colorectal *in siu* or invasive carcinoma.**

| FDRs with carcinoma^a^ | Relatives with polyp | Frequency of polyp diagnosis | No. of observed CRC patients | SIR^b^ | 95% CI |
| --- | --- | --- | --- | --- | --- |
| 0 | 0 FDR/SDR | NA | 5,889 | Reference | Reference |
| 1 | 0 FDR/SDR | NA | 799 | **1.7** | 1.5-1.8 |
|  | ≥1 FDR/SDR | ≥1 | 300 | **3.5** | 3.2-4.0 |
|  | 1 FDR only | 1 | 113 | **3.0** | 2.4-3.6 |
|  |  | ≥2 | 47 | **4.9** | 3.6-6.5 |
|  | ≥2 FDRs | 1 | 11 | **4.9** | 2.6-8.8 |
|  |  | ≥2 | 26 | **15.3** | 10.0-22.4 |
|  | 1 SDR only | 1 | 45 | **2.1** | 1.5-2.8 |
|  |  | ≥2 | 16 | **3.6** | 2.0-5.8 |
|  | ≥2 SDRs | 1 | 3 | 2.2 | 0.4-6.0 |
|  |  | ≥2 | 3 | **6.2** | 1.3-17.9 |
| ≥2 | 0 FDR/SDR | NA | 75 | **2.6** | 2.0-3.2 |
|  | ≥1 FDR/SDR | ≥1 | 64 | **7.7** | 5.9-9.9 |
|  | 1 FDR only | 1 | 17 | **5.4** | 3.1-8.6 |
|  |  | ≥2 | 13 | **11.6** | 6.2-19.9 |
|  | ≥2 FDRs | 1 | 2 | 6.4 | 0.9-23.1 |
|  |  | ≥2 | 8 | **26.4** | 11.4-52.0 |
|  | 1 SDR only | 1 | 7 | **3.9** | 1.6-8.1 |
|  |  | ≥2 | 1 | 2.5 | 0.3-14.1 |
|  | ≥2 SDRs | 1 | 0 | NA | NA |
|  |  | ≥2 | 7 | **8.4** | 3.4-17.4 |

^a^Including colorectal *in situ* and invasive carcinoma (Stage 0 to IV).

^b^SIR adjusted for age, sex, calendar year, region, history of diabetes mellitus, and socioeconomic status, and inpatient and outpatient visits to specialty clinics due to obesity, alcoholism and chronic obstructive pulmonary disease. Bold SIR indicates statistically significant (95%CIs did not include 1.00).

Abbreviations: CI, confidence interval; CRC, colorectal cancer; EOCRC, early-onset colorectal cancer; FDR, first-degree relative; SDR, second-degree relative; SIR, standardized incidence ratio; NA, not applicable.


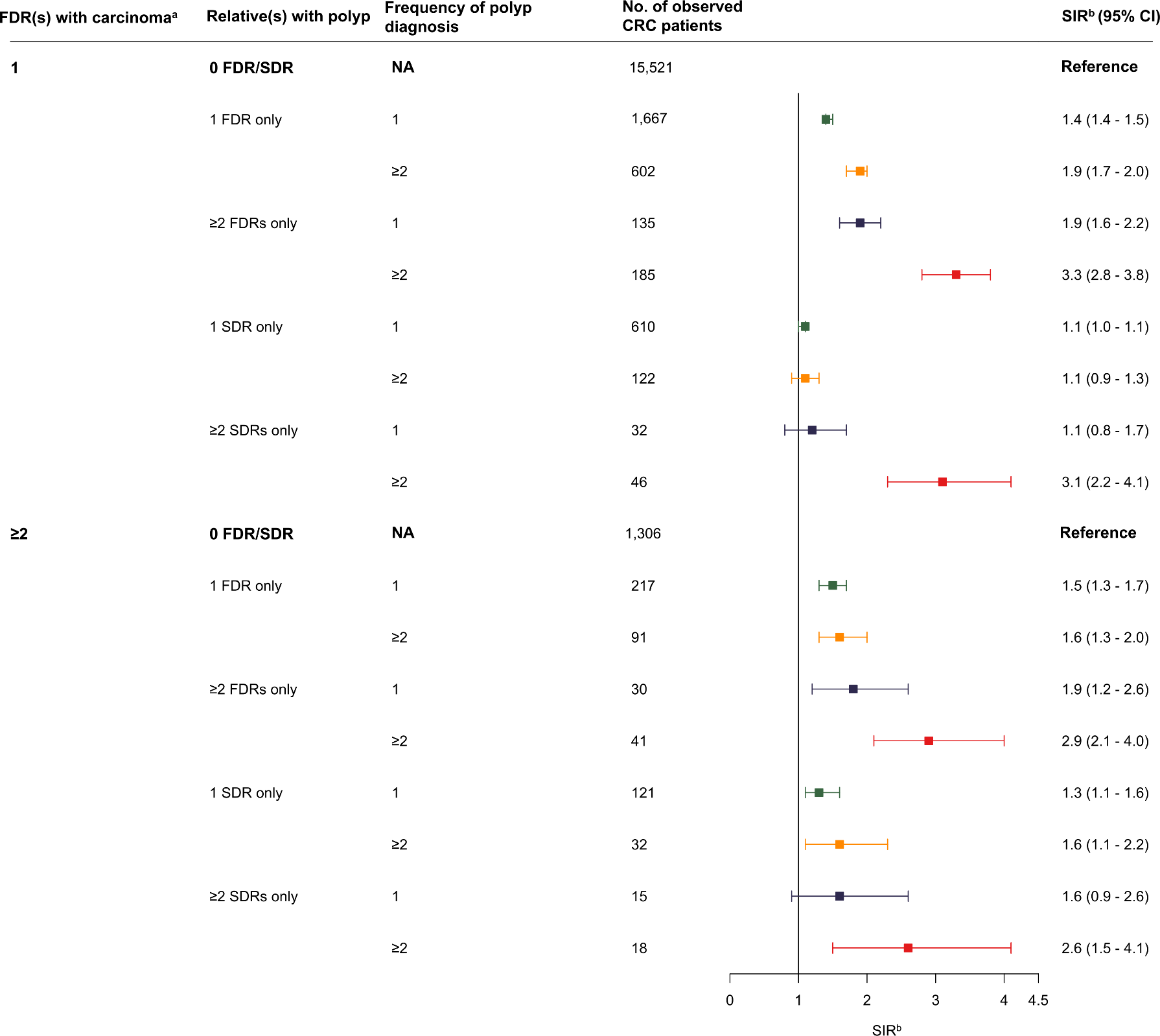


**Supplementary Figure S1. Risk of CRC in individuals with family history of both colorectal polyp and carcinoma** **compared with individuals with family history of colorectal carcinoma alone.**

^a^Including colorectal *in situ* and invasive carcinoma (Stage 0 to IV).

^b^SIR adjusted for age, sex, calendar year, region, history of diabetes mellitus, and socioeconomic status.

Abbreviations: CI, confidence interval; CRC, colorectal cancer; FDR, first-degree relative; SDR, second-degree relative; SIR, standardized incidence ratio; NA, not applicable.


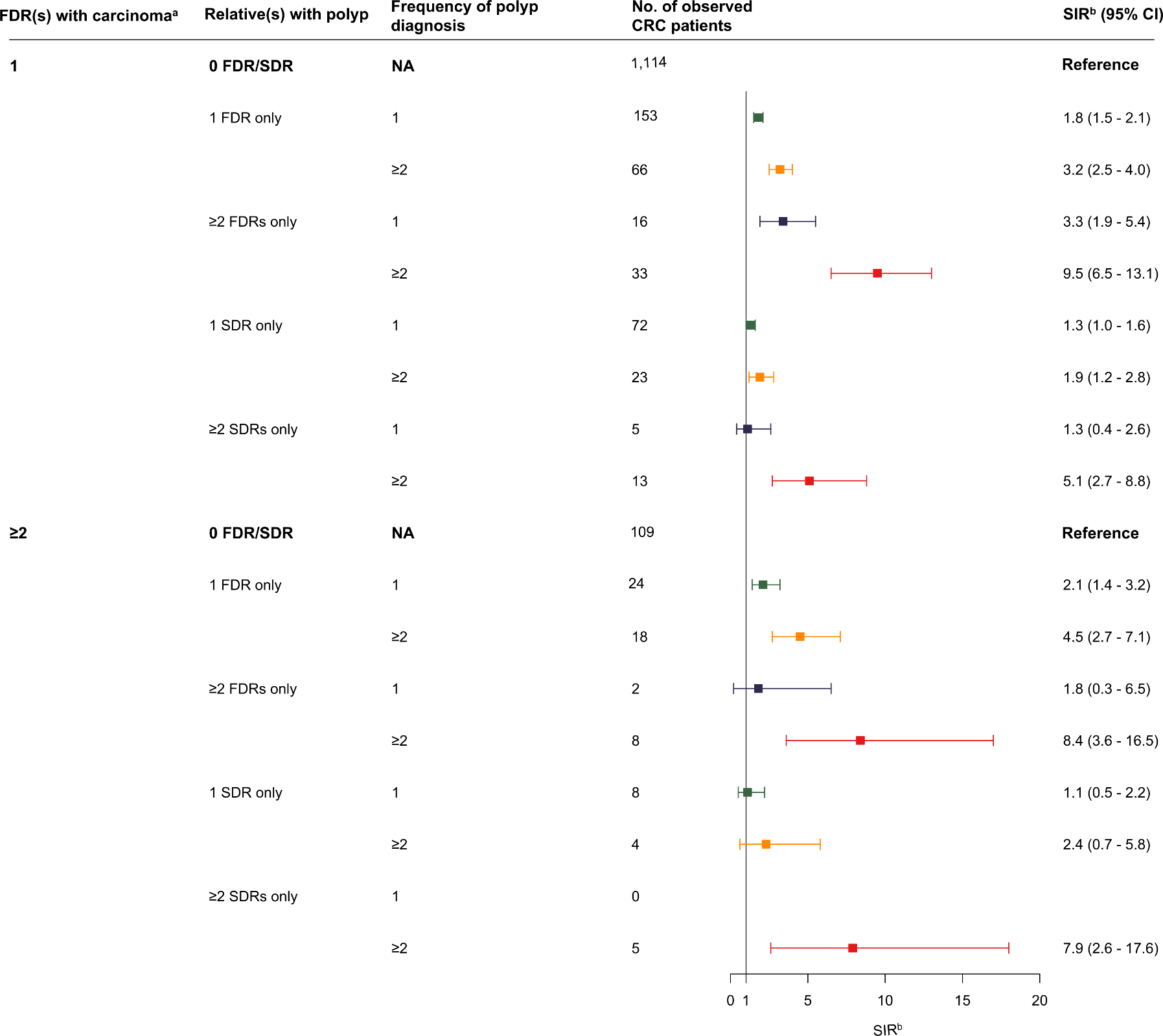


**Supplementary Figure S2. Risk of EOCRC in individuals with family history of both colorectal polyp and carcinoma compared with individuals with family history of colorectal carcinoma alone.**

^a^Including colorectal *in situ* and invasive carcinoma (Stage 0 to IV).

^b^SIR adjusted for age, sex, calendar year, region, history of diabetes mellitus, and socioeconomic status.

Abbreviations: CI, confidence interval; CRC, colorectal cancer; EOCRC, early-onset colorectal cancer; FDR, first-degree relative; SDR, second-degree relative; SIR, standardized incidence ratio; NA, not applicable.
